# Mantle heterogeneity influenced Earth’s ancient magnetic field

**DOI:** 10.1038/s41561-025-01910-1

**Published:** 2026-02-03

**Authors:** A. J. Biggin, C. J. Davies, J. E. Mound, S. J. Lloyd, Y. E. Engbers, D. Thallner, A. T. Clarke, R. K. Bono

**Affiliations:** 1 Geomagnetism Laboratory, Department of Earth, Ocean and Ecological Sciences, University of Liverpool, Liverpool, UK; 2 School of Earth and Environment, University of Leeds, Leeds, UK; 3 Electromagnetic Signatures and Propagation, Netherlands Organisation for Applied Scientific Research (TNO), The Hague, The Netherlands; 4 Department of Geological Sciences, University of Florida, Gainesville, FL USA; 5 Department of Earth, Ocean and Atmospheric Science, Florida State University, Tallahassee, FL USA

**Keywords:** Palaeomagnetism, Core processes, Geomagnetism, Geodynamics

## Abstract

Heat flowing from the core to the mantle drives the geodynamo that produces Earth’s global magnetic field. Palaeomagnetic measurements record the behaviour of this field through time and have the potential to inform us about deep Earth structures and dynamics on either side of the core–mantle boundary. In practise, insights have proved difficult to obtain because of the limited spatiotemporal resolution of palaeomagnetic records and uncertainties in how to interpret them. Here we use palaeomagnetic datasets and models alongside numerical simulations of the geodynamo to show that certain observed characteristics of ancient magnetic field behaviour are uniquely or preferentially reproduced in the presence of strong lateral variability in core–mantle heat flux. Our findings suggest that strong contrasts in the spatial pattern of the temperature gradients and/or thermal conductivity of the lowermost mantle that are linked, today, to seismologically observed structures, have influenced the geodynamo for at least the last few hundred million years. The identified palaeomagnetic signatures provide a new means to constrain the properties and time evolution of the core–mantle boundary. Furthermore, our insights into how thermal heterogeneity at the base of the mantle can break the axial symmetry of the time-averaged magnetic field may help resolve longstanding palaeogeographic controversies.

## Abstract (web summary)

Earth’s core dynamo, which produces the magnetic field, may have been influenced by spatial variations in heat flux across the core–mantle boundary, according to combined palaeomagnetic datasets and geodynamo simulations.

## Main

The lowermost mantle acts as the cold sink for the geodynamo heat engine and therefore exerts a first-order control on the geomagnetic field that is generated in Earth’s outer core. Seismically, this region is highly heterogeneous^1^ and characterized, in the bottom few hundred km, by a ‘recumbent Y20 pattern’ of two large low-velocity provinces (LLVPs) located in antipodal positions close to the equator separated by a pole-to-pole girdle of faster than average seismic velocities^2,3^. This region probably contains substantial lateral variations in temperature^4^ with the LLVPs hotter than the surrounding girdle^5^. Considerable variability in core–mantle heat flux is implied with geodynamical investigations supporting peak-to-peak differences of more than twice the average^6^. The long-term stability of LLVPs is a controversial topic^7,8^ with major implications for the mantle’s properties, dynamics and evolution through time.

Large lateral variations in core–mantle heat flux are expected to influence flow of liquid iron alloy in the underlying core^9,10^ and in the geomagnetic field that is generated there. Palaeomagnetic records preserve this signal and therefore could constrain the thermal properties of the lowermost mantle and its evolution through time but this is non-trivial to unravel^11^. Previous investigations have tended to focus on time intervals shorter than 100 kyr (refs. ^11–20^) or to use core–mantle heterogeneity to explain changes in palaeomagnetic field behaviour over 100s of Myr (refs. ^13,21,22^). The former approach relies on a snapshot of geological time while the latter relies on poorly understood aspects of palaeomagnetic behaviour. Here we focus initially on extracting the most robust observations of palaeomagnetic behaviour from the last few tens of Myr that are relevant to thermal core–mantle interaction. By combining observation-based field models, palaeomagnetic data compilations and numerical simulations of the geodynamo, we provide substantial new evidence for signatures of heterogeneous core–mantle heat flow in ancient palaeomagnetic records.

## Palaeomagnetic observations from 0 to 23 Ma

We aim to identify robust, salient features of the recent palaeomagnetic field that can be compared to the predictions of dynamo simulations focusing on palaeomagnetic directions that are more common and reliable than intensities. These describe palaeosecular variation (PSV) through the angular dispersion of virtual geomagnetic poles (VGPs)^23^ and are used in global models of the time-averaged field (TAF)^24^. Long-term stability of PSV revealed by recently updated records from the last 265 Myr (refs. ^25–29^) provides a strong constraint on geodynamo simulations. TAF models, by contrast, span only the last 23 Myr but may elucidate persistent geomagnetic field structures providing signatures of the mantle’s influence on the geodynamo^30^.

We pay particular attention to longitudinally varying signals in both PSV and the TAF, which may provide a signature of strong thermal heterogeneity at the base of the mantle forcing the geodynamo^11^.

**Fig. 1:**
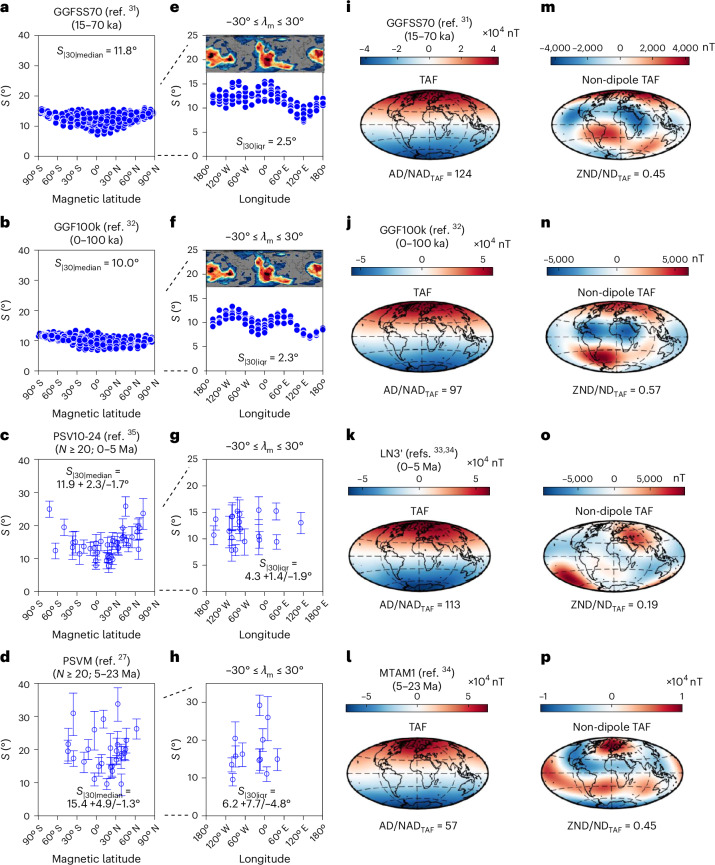
PSV and TAF defined for four time periods by palaeomagnetic models and datasets. **a**–**h**, *S* is VGP angular dispersion plotted against magnetic latitude (**a**–**d**) and against geographic (palaeo)longitude using only those estimates from magnetic latitudes, *λ*_m_ ≤ ± 30° (**e**–**h**). **i**–**p**, The magnitude of the radial component of the time-averaged field at Earth’s surface (truncated to degree and order 4) is plotted in **i**–**l** and the same, after the dipole field is removed, is shown in **m**–**p**. The four summary criteria are defined in the text. For reference, panels **e** and **f** show global vote maps of lower-than-mean S-wave velocities calculated from 18 tomographic models at a depth of 2,850 km using the SubMachine website^38^. Error bars were calculated using 10,000 bootstrap resamples with replacement. Basemaps in **i**–**p** generated with M_Map^54^. Source data

PSV and TAF structure evident in models and datasets derived from four intervals in the last 23 Myr (Methods) are summarized (Fig. 1). Palaeomagnetic field models^31,32^ cover intervals 15–70 ka (kiloannum) and 0–100 ka, whereas palaeomagnetic datasets and time-averaged field models^33,34^ are from volcanic rocks of ages 0–5 Ma (ref. ^35^) and 5–23 Ma (ref. ^27^), respectively.

We characterize PSV by the angular dispersion of virtual geomagnetic poles (*S*; Methods) after excluding outlier VGPs^36^ associated with excursions and reversals. Values of *S* at low magnetic latitudes provide proxies for the average instantaneous axial dipole dominance^37^. We parameterize this using the median (*S*_|30|median_) and interquartile range (*S*_|30|iqr_) of values of *S* obtained at sites located between magnetic latitudes 30° N and 30° S (Methods). *S*_|30|median_ and *S*_|30|iqr_ in the 0–5 Ma intervals cover narrow ranges from 10.2 to 14.2° and 2.3 to 5.7°, respectively, while the 5–23 Ma interval has larger uncertainties (Fig. 1a–d).

The source of the variance that is quantified by *S*_|30|iqr_ is apparent from plots of *S*, drawn from the two time-varying models at low-latitude locations, against longitude (Fig. 1e,f). A double hump in *S* appears with peaks at ~100° W and ~ 40° E with one minimum between them at ~20-50° W and a more pronounced minimum at ~130° E. Seismic heterogeneity of the lowermost mantle^38^ is dominated by spherical harmonic degree 2 (Fig. 1e,f). Such a pattern matches the wavelength of the modulation in *S*. Although caution should be exercised in interpreting individual features associated with any such models^39^, the striking correlation between the models and the seismic tomography suggests that heterogeneity in the lowermost mantle could be modulating PSV. If correct, some semblance of this longitudinally varying pattern should persist in datasets from millions of years ago because the mantle would be largely unchanged. A direct test of this hypothesis using data from 0 to 5 Ma and 5–23 Ma is inconclusive (Fig. 1g,h) because of the large uncertainties on *S*. We show later, however, that variance in *S* at low latitudes, preserved in records spanning longer timescales, provides indirect support for long-lived azimuthal asymmetry in the palaeomagnetic field.

TAF models are dominated, at the Earth’s surface, by an axial dipole (Fig. 1i–l), which we quantify using a ratio of the Lowes Power (AD/NAD_TAF_; Methods). Note that AD/NAD_TAF_ is derived from the spectrum of the averaged field and does not represent the average of many time-instantaneous ratios as calculated elsewhere^37^. Interesting features emerge in maps of the TAF with the dipole components removed (Fig. 1m–p). Boundary control of the geodynamo need not imply locking of the field, and the repeatability of individual features between time periods is debatable. Nevertheless, a robust characteristic is that all models have substantial power in both zonal (varying only with latitude) and non-zonal families of spherical harmonic terms. We quantify this using another ratio (ZND/ND_TAF_; Methods) and find that only 0.19 to 0.57 of power in the non-dipole part of the TAF field is azimuthally symmetric. For comparison, the geomagnetic field in 2015^40^ exhibited a comparable ZND/ND value of 0.23 (but a much lower AD/NAD value of 10 compared to AD/NAD_TAF_ values > 50 because the latter are time averages).

### Comparison with outputs of numerical dynamo simulations

We initially consider magnetic field behaviour output by 31, mostly new, numerical dynamo simulations forming six distinct groups within which only the input Rayleigh number (Ra) was varied (Methods and Supplementary Table 1). Two of these groups (nine simulations) were thermally driven with uniform heat flux imposed at the inner and outer boundaries. Four groups comprised thermal or thermochemically driven simulations in which a strongly heterogeneous pattern of heat flux was imposed on the outer boundary. A tomographic model^1^ provided the basis for this pattern characterized by two antipodal and equatorial negative flux anomalies beneath Africa and the Pacific representing the LLVPs. The q* parameter defines the magnitude of this heterogeneity:

1 $${q}^{* }=\frac{{q}_{\max }-{q}_{\min }}{{q}_{\mathrm{ave}}},$$

where *q*_max_, *q*_min_ and *q*_ave_ define the maximum, minimum and average heat flux, respectively. In three heterogeneous groups, q* was set to 2.3; in the other, q* was set to 5.0.

**Fig. 2:**
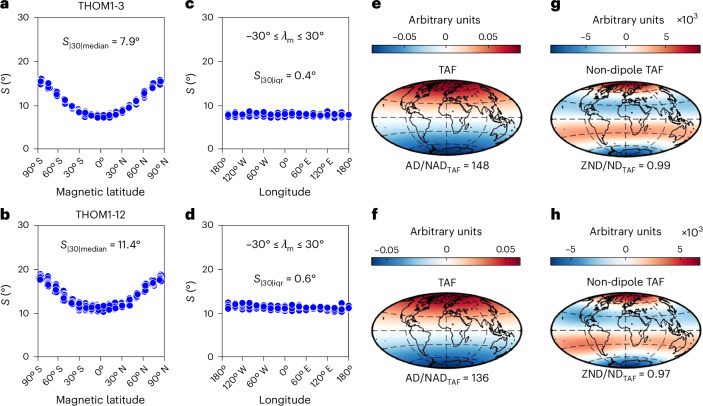
Examples of PSV and TAF output from dynamo simulations run with homogeneous outer boundary conditions. Supplementary Table 1 provides input parameters of models, and Fig. 1 and Methods provide descriptions of panels and output parameters. Outputs from all homogeneous models are shown in Supplementary Figs. 1 and 4. Basemaps in **e**–**h** generated with M_Map^54^. Source data

**Fig. 3:**
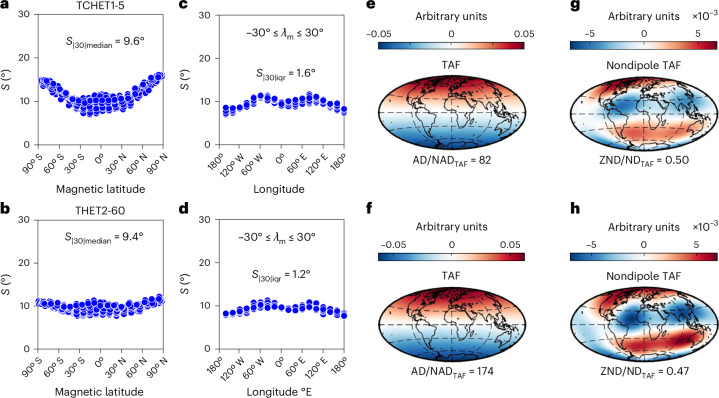
Examples of PSV and TAF output from dynamo simulations run with heterogeneous outer boundary conditions. Supplementary Table 1 provides input parameters of models, and Fig. 1 and Methods provide descriptions of panels and output parameters. Outputs from all heterogeneous models are shown in Supplementary Figs. 2–4. Basemaps in **e**–**h** generated with M_Map^54^. Source data

**Fig. 4:**
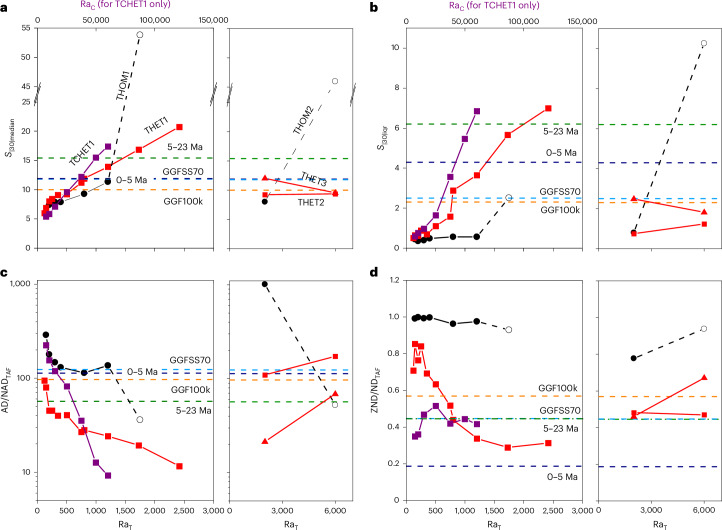
Summary of outputs from all geodynamo simulations and comparison to palaeomagnetic constraints using four terms defined in main text. **a**–**d**, In each of the four panels, lines connect simulations from the same groups (names given in **a**) within which only Ra_T_ (and Ra_C_ in TCHET1) were varied. Simulations shown on left of each panel used parameters *E* = 3 × 10^−4^, Pr = 1 and Pm = 3 (and Pr_C_ = 10 for TCHET1) whereas those on the right used *E* = 2 × 10^−5^, Pr = 0.2 and Pm = 1 (Supplementary Table 1). Black circles denote thermally driven simulations run with homogeneous boundary conditions (q* = 0; unfilled circles indicate simulations outputting a multipolar magnetic field). Red symbols denote thermally driven simulations run with heterogeneous boundary conditions (squares where q* = 2.3; triangles where q* = 5.0). Purple symbols denote thermochemically driven simulations run with heterogeneous boundary conditions (q* = 2.3). Note that the Ra_T_ axes apply to all plots whereas the Ra_C_ axes apply only to TCHET1.

Input parameters set for each simulation group (Methods and Supplementary Table 1) produced values of *S*_|30|median_ and AD/NAD_TAF_ that are consistent with palaeomagnetic values across a range of input values of Ra (Figs. 2–4). Homogeneously forced simulations were slightly more directionally stable (having lower values of *S*_|30|median_) with slightly higher values of AD/NAD_TAF_.

**Extended Data Fig. 1:**
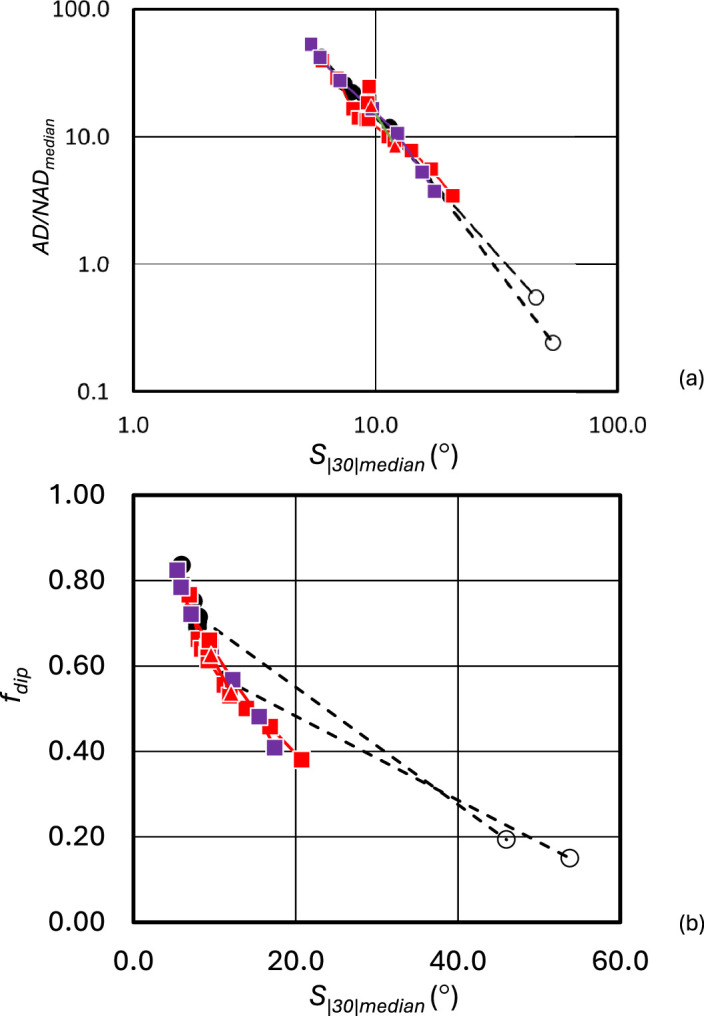
Axial dipole dominance at Earth’s surface and dipole dominance at the core-mantle boundary predicted by *S*_*|30|median*_ output by geodynamo simulations. Black circles represent simulations with homogeneous boundary conditions (unfilled indicates multipolar morphology). See Fig. 4 for a full definition of symbols.

**Extended Data Fig. 2:**
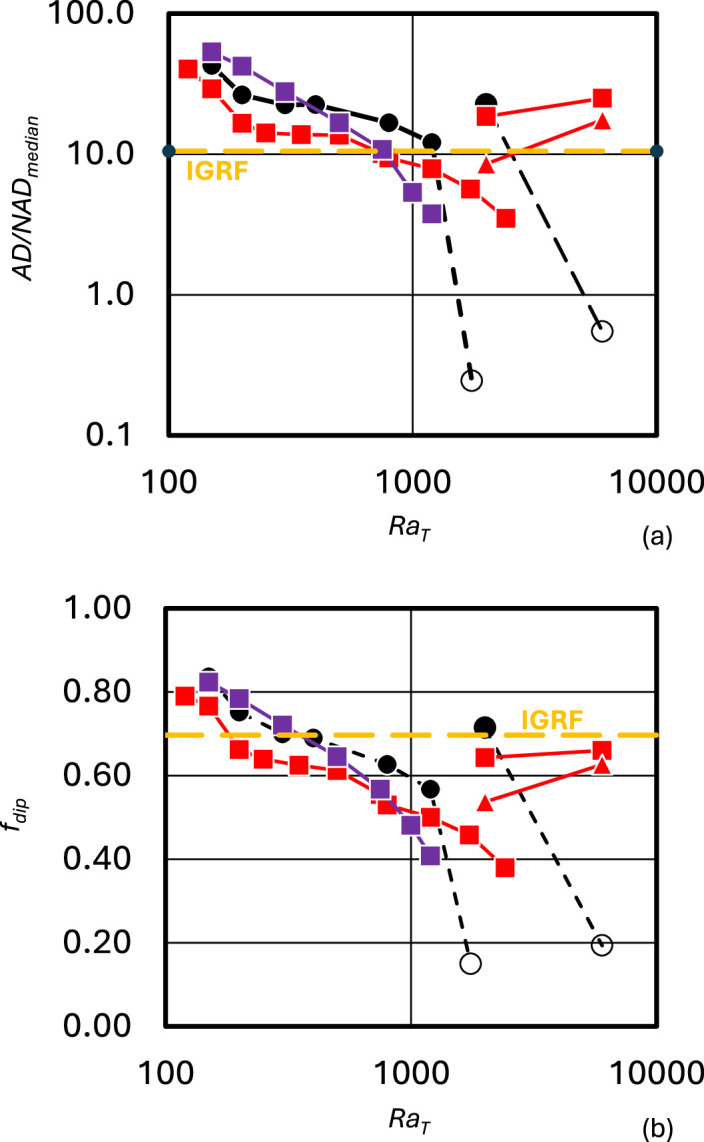
Axial dipole dominance at Earth’s surface^33^ and dipole dominance at the core-mantle boundary^60^ output by geodynamo simulations as a function of the thermal Rayleigh number, *Ra*_*T*_. Values for IGRF model^36^ (1900-2015) are shown for reference. See Fig. 4 caption for definition of symbols.

At high values of Ra, outputs of *S*_|30|median_ from homogeneous simulations jump from < 12° to > 45° (Fig. 4a and Supplementary Figs. 1 and 4) and the time-instantaneous field morphology shifts to a multipolar state (Extended Data Figs. 1 and 2) not appropriate for the Earth outside of reversal transitions and excursions. This was not observed in heterogeneous simulations (Fig. 4a and Supplementary Figs. 2 and 3); the strong thermal heterogeneity imposed on the outer boundary prevented these crossing the multipolar transition^41^.

Heterogeneous simulations exhibited longitudinally varying values of *S* with a degree-2 wavelength (Fig. 3 and Supplementary Figs. 2 and 3) showing a general similarity with the pattern obtained from the observational models (Fig. 1). This low-latitude modulation of *S* increases *S*_|30|iqr_ rendering heterogeneous simulations compatible with the observational constraints. Dipole-dominated homogeneous simulations, by contrast, exhibit axisymmetric PSV and *S*_|30|iqr_ values that are too low (Fig. 4b).

Comparable values of AD/NAD_TAF_ notwithstanding (Fig. 4c), the nature of the time-averaged non-dipole fields differ profoundly between the homogeneous and heterogeneous simulations. The ${g}_{3}^{0}$ (axial octupole) term dominates the non-dipole time-averaged field of every homogeneous simulation (Fig. 2 and Supplementary Figs. 1 and 4) while other (zonal and non-zonal) terms persist in many heterogeneous simulations, particularly at higher values of Ra. These break the negative band of non-dipole field in the Northern Hemisphere into two flux patches and focus the southern positive patch in the Atlantic hemisphere while pushing it polewards. Similar patterns are seen in the nondipolar TAF of the two palaeomagnetic models (Fig. 1m,n) although the details differ. ZND/ND_TAF_ values (Fig. 4d) demonstrate that the heterogeneous simulations, particularly those with thermochemical forcing, can clearly satisfy the palaeomagnetic constraint of symmetry breaking while homogeneous simulations cannot.

In summary, all groups of simulations appear capable of being tuned to meet the constraints of *S*_|30|median_ and/or AD/NAD_TAF_ although this can be achieved more readily in those with heterogeneous boundary conditions. By contrast, groups with homogeneous outer boundary conditions are entirely incapable of meeting the criteria *S*_|30|iqr_ and ZND/ND_TAF_ while maintaining dipole-dominated fields (Fig. 4). The key finding here is that independent of other input parameters, q* > 0 is a requirement for observation-based criteria to be met by these diverse simulations. Tuning of q* (> 0) and other input parameters may allow simultaneous satisfaction of our four constraints alongside other published criteria^42,43^, but that exceeds the scope of this study.

## Palaeomagnetic datasets derived from deeper in geological time

The high viscosity of the lowermost mantle implies that core–mantle heat flow should vary very slowly through time. Therefore, signatures of the influence of mantle spatial heterogeneity on the geodynamo may also be evident in the older palaeomagnetic field.

**Fig. 5:**
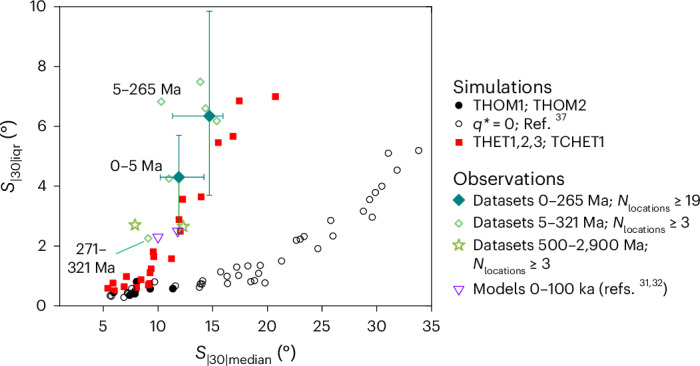
Comparison of PSV terms measured using palaeomagnetic datasets, field models and geodynamo simulations run with homogeneous and heterogeneous heat flux conditions on the outer boundary. Unfilled circles are simulations (with homogeneous boundary conditions and present-day inner core size) analysed in a previous study^37^. The 5–265 Ma dataset combines five studies (shown as unlabelled diamonds; Extended Data Table 1) to allow uncertainties to be estimated. The *S*_|30|median_ axis was truncated at 35° for clarity because simulations beyond this exhibited multipolar fields. Error bars were calculated using 10,000 bootstrap resamples with replacement.

No models of the time-averaged field have yet been made for intervals prior to 23 Ma but records of PSV similar to those shown in Fig. 1b,c have been produced from rocks with ages up to 2,900 Ma (refs. ^25–29,44^) (Extended Data Table 1). The 0–5 Ma dataset has sufficient low-latitude estimates of *S* to allow meaningful uncertainty limits to be obtained for *S*_|30|median_ and *S*_|30|iqr_. Five individual published datasets spanning the age range 5–265 Ma were combined (Fig. 5) to obtain a further robust dataset for more ancient times. This is justified by the observed similarity of PSV records spanning 0–265 Ma (ref. ^28^).

Regardless of size or age, all palaeomagnetic datasets plot, together with the palaeomagnetic field models for 0–100 ka, in a region of *S*_|30|median_–S_|30|iqr_ space (Fig. 5) that is uniquely populated by simulations run here using heterogeneous outer boundary conditions. *S*_|30|median_ values form a narrow band between ~9 and 16° and *S*_|30|iqr_ values range from ~2 to 9°. The former constraint is met more readily by heterogeneous than homogeneous simulations. The latter is not satisfied, simultaneously with the *S*_|30|median_ constraint, by homogeneous simulations from this or a previous study^37^ (Fig. 5).

**Extended Data Fig. 3:**
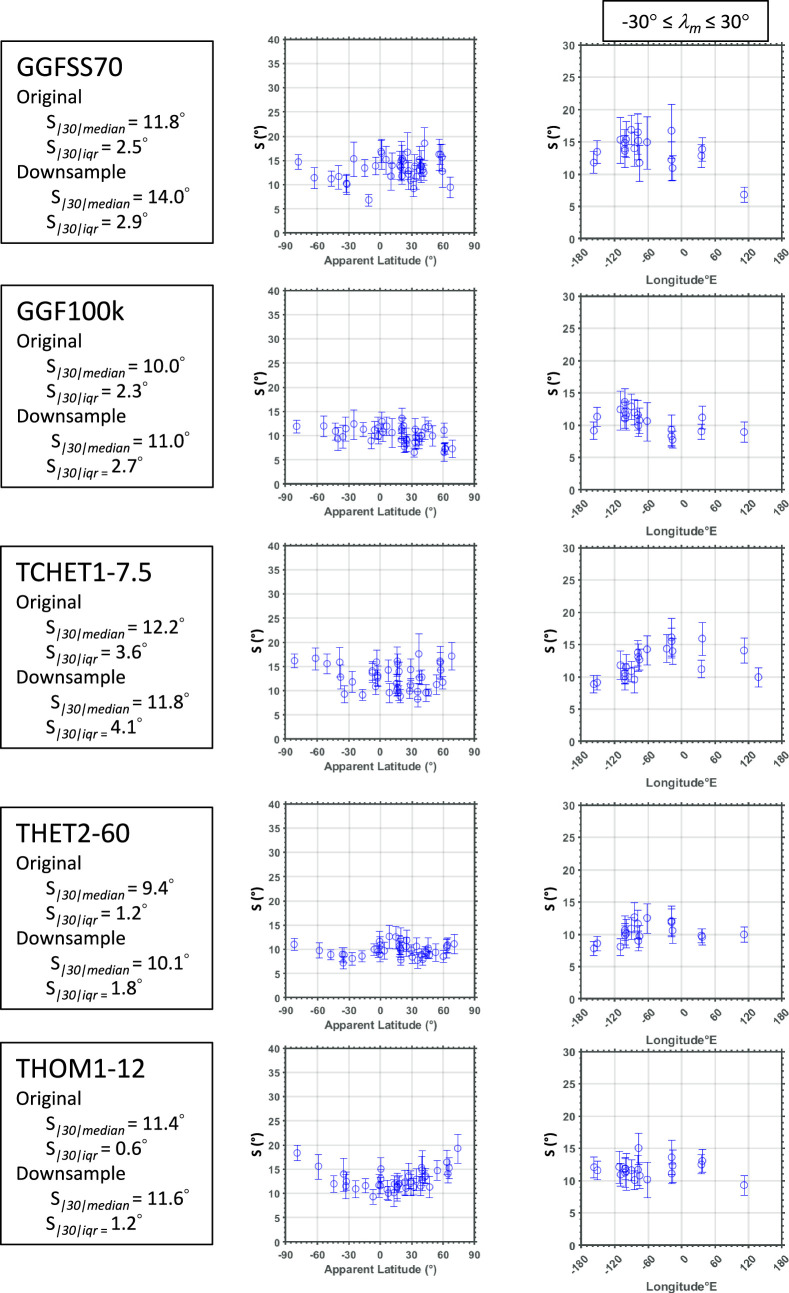
Examples of PSV plots calculated from models and simulations that were randomly downsampled in time according to the geographic distribution of sites in the PSV10-24 dataset. For details of simulations, see Table S1. Error bars were calculated using 10,000 bootstrap resamples with replacement.

**Extended Data Fig. 4:**
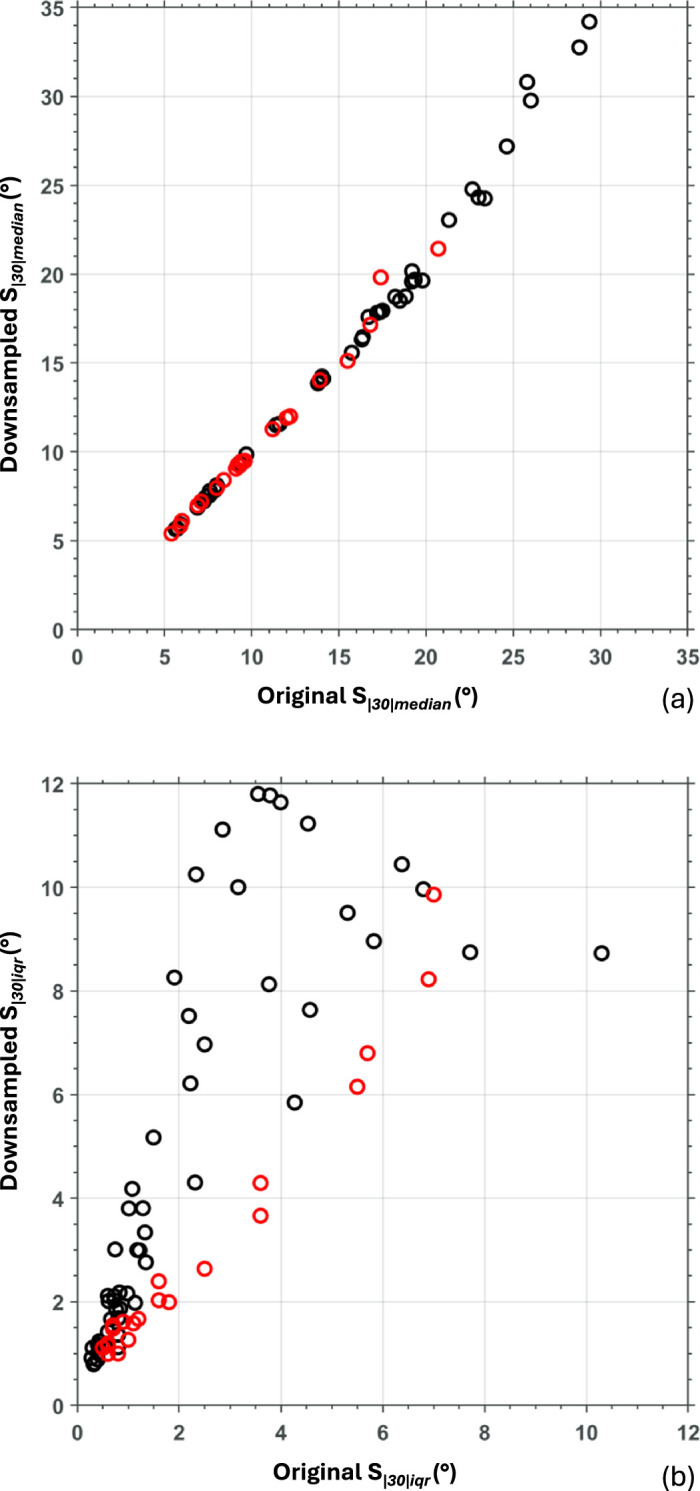
Comparisons of example output PSV statistics of simulations before and after downsampling at 28 random locations with 31 measurements made at each (values chosen to represent 5-265 Ma). Black (red) circles represent homogeneous (heterogeneous) simulations. Source data

**Extended Data Fig. 5:**
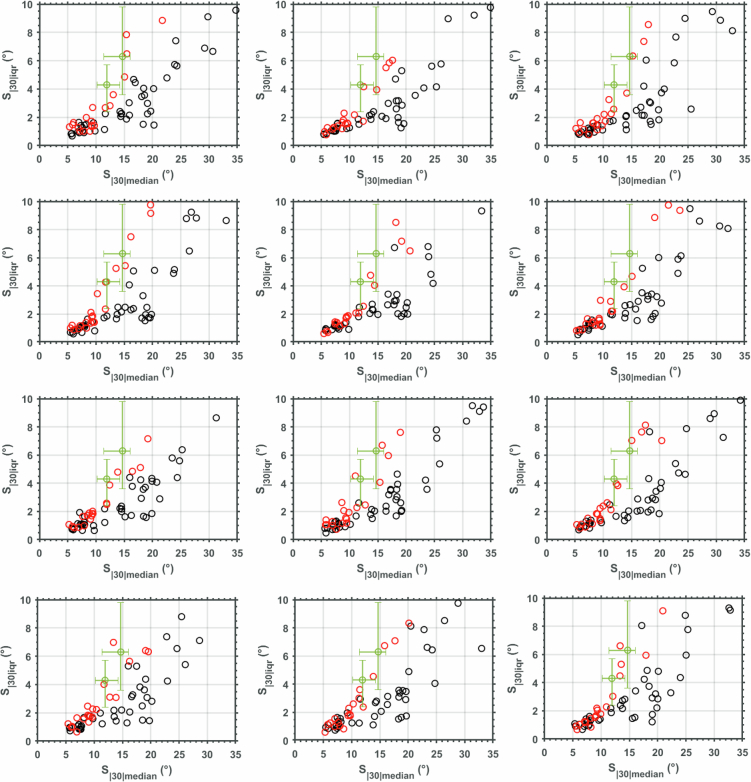
PSV properties of 12 random downsamples of simulations with homogeneous (black) and heterogeneous (red) outer boundary conditions. Each simulation (circle) was downsampled used 31 random time steps (each representing a palaeomagnetic direction) at 28 locations. These numbers were chosen to represent the availability of PSV data 5-265 Ma. Green points refer to 0-5 Ma and 5-265 Ma datasets shown in Fig. 5 and detailed in Extended Data Table 1. Error bars shows 95% uncertainties calculated from 10,000 bootstrap resamples with replacement.

We consider it likely that any long-wavelength signal in the longitudinal dependence of *S* (Fig. 1e,f) in the palaeomagnetic datasets (Fig. 1g,h) is obscured by additional variance contributed by the small number of both sampling localities on the globe and palaeomagnetic directions obtained at each site (Extended Data Table 1). This is supported by the observation that when models and simulations were randomly downsampled using the spatial distribution of data from 0 to 5 Ma, the longitudinal signal became difficult to discern (Extended Data Fig. 3). By the same token, however, the downsampling could also exaggerate values of *S*_|30|iqr_ (especially those from homogeneous simulations; Extended Data Fig. 4). This questions whether high values of *S*_|30|iqr_ observed in the palaeomagnetic datasets result from their sparse distribution and do not require mantlel heterogeneity to explain. We tested this by running 12 random downsamples of every simulation shown in Fig. 5 (Extended Data Fig. 5). While some blurring between regions of *S*_|30|median_–S_|30|iqr_ space occupied by homogeneous vs heterogenous simulations occurred, the outputs from the former clearly remain incompatible with the palaeomagnetic datasets. On this basis, and because homogeneous simulations cannot reproduce non-zonal structures apparent in models of the 0–23 Ma field, we conclude that a signal of lateral variations in core-mantle heat flux is present in palaeomagnetic data from that interval and probably since 265 Ma. Lower values of *S*_|30|iqr_ obtained from still-older datasets (spanning 271–2,900 Ma) makes it less certain that their incompatibility with homogeneous simulations (Fig. 5) is genuine.

## Implications for the geodynamo and core–mantle heterogeneity

**Extended Data Fig. 6:**
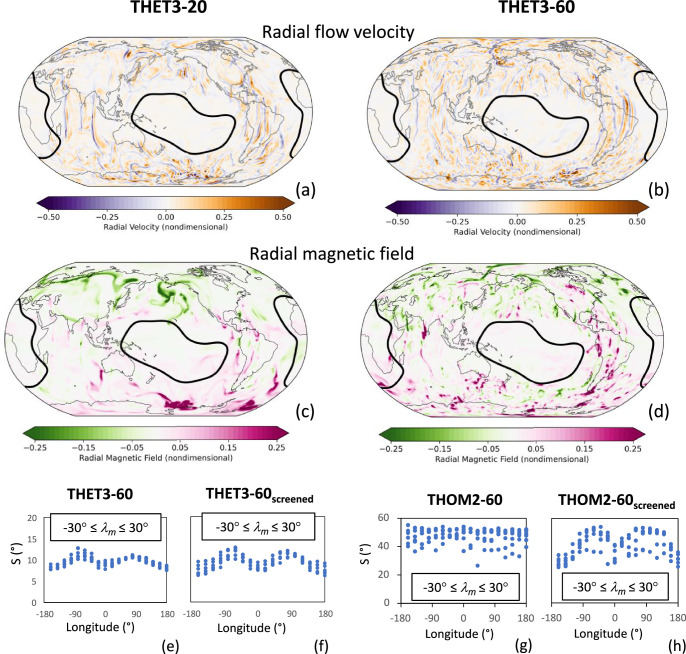
Flow and field output by two simulations under strong heterogeneous forcing and results of a magnetic screening analysis. Snapshots of radial flow (**a**, **b**) and magnetic field (**c**, **d**) at the top of the core in two THET3 (q* = 5.0) simulations. The position of the 1% slow contour from the seismic model^1^ is outlined in black and shows the location of the large low-velocity provinces (LLVPs) associated with a minimum in heat flux. The radial flow was taken 200 km below the core-mantle boundary (CMB) while the magnetic field was taken at the CMB. In all cases, the projection is centred on 180°E. The LLVPs clearly suppresses flow and magnetic field in the shallow core. This imposes large-scale order on the field at low latitudes on the CMB. In (**e**–**h**) we show the effect of purely the magnetic screening (see Methods) under the LLVPs on the PSV outputs of simulations run with different thermal conditions imposed on the outer boundary. Where strong thermal heterogeneity was imposed (**e**, **f**), the additional screening had little effect suggesting intrinsic screening had already occurred. Where homogeneous outer boundary conditions were imposed (**g**, **h**), the screening suppressed low (±30°) latitude VGP dispersion and imparted a longitudinal dependence rendering the outputs more Earth-like. Nevertheless, the screening effect alone was insufficient to render the PSV identical in these cases indicating that strong thermal heterogeneity impacts on the dynamo process itself as well as screening the field that is output. Basemaps in **a**–**d** generated with Matplotlib^76^ and Cartopy^77^.

The mechanism by which strong thermal heterogeneity influences the structure and stability of the geodynamo simulations would need a dedicated study to elucidate in detail. It probably combines a magnetic field screening effect at the surface of the spherical shell with a more complex mechanism involving the underlying dynamo process. Regional suppression of convection occurs beneath areas of low heat flux, which correspond to LLVPs in seismic tomographic models^10^. Partial stratification produces a stabilizing skin effect diffusing small-scale fields and allowing the dipole to remain dominant^41^. Both convection and magnetic induction are suppressed beneath areas of low heat flux in our simulations. We find (Methods) that the latter helps to make the PSV appear more like that observed in the palaeomagnetic field models (Extended Data Fig. 6). Thermal heterogeneity can also influence the stability of the dipolar state by altering the structure of the zonal flow^45^. In a subset of our simulations^11^, we find that the latitudinal temperature variation induced by the boundary heterogeneity (comprising a hot equatorial region and cooler high latitudes) drives thermal winds that reinforce the persistent westward zonal flow that has been found to correlate with a stable dipolar state^45^. We expect such an effect to be present as far back in geological time as the broad structure of the LLVPs remains similar to that observed at present.

Our combined analysis of palaeomagnetic models, datasets and geodynamo simulations substantially strengthens the case that heterogeneity in core–mantle heat flux affects the long-term behaviour of the geodynamo. This, in turn, implies that seismic heterogeneity observed at the base of the mantle corresponds, at least in part, to sizable variations in temperature gradients and/or thermal conductivity in that region. Our findings are that probably through the generation of anomalous structures in the uppermost core, this thermal heterogeneity: (1) adds longitudinal structures to palaeomagnetic secular variation, (2) does the same for the time-averaged field and (3) may be important for maintaining the dipole dominance of the time-instantaneous field. Limitations include the availability of current palaeomagnetic data and the difficulty of exploring a wide region of parameter space at extreme conditions using direct numerical simulation of the geodynamo. Nevertheless, potential biases have been sought and mitigated against as far as possible and our findings are testable, refinable and extendable. Larger palaeomagnetic datasets will reduce uncertainties allowing better comparisons with the outputs of dynamo simulations that explore a wider parameter range. Compromises are necessary when modelling core dynamics over the long timescales required for comparison to palaeomagnetic data. Nevertheless, there are already reasons to expect that the germane features of our simulations can be extrapolated to Earth’s core conditions. Our most extreme simulations do access rapidly rotating, strong-field conditions that are expected to be applicable to Earth’s core^46,47^. Furthermore, the large-scale dynamics induced by thermal heterogeneity close to the upper boundary are expected to be independent of viscosity and inertia^48^ (both very small in Earth) and resilient to disruption from smaller-scale convection at greater depths^46,49,50^. Our other simulations, while further from this regime, also satisfy palaeomagnetic constraints while being integrated over considerably longer intervals and, crucially, exhibit the same key dependence on q*, strengthening the findings. Beyond palaeomagnetism and geodynamo simulation, these findings invite seismology to probe the structure of the uppermost core and test whether mantle heterogeneity produces persistent structures as inferred here^10^.

**Extended Data Fig. 7:**
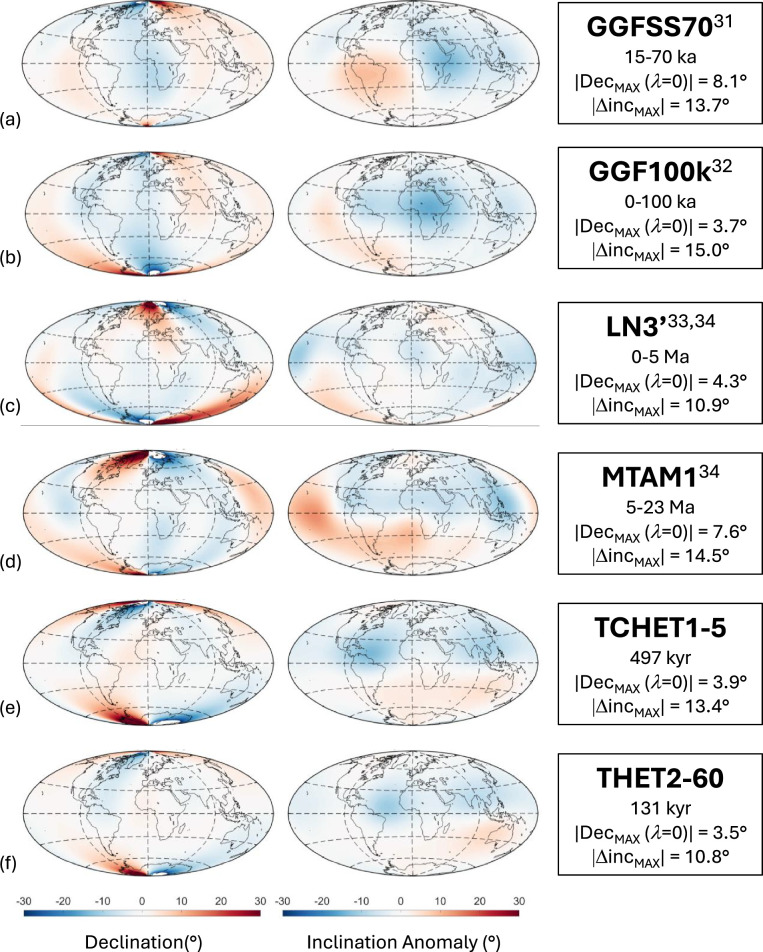
Declination and Inclination anomalies (that is deviations from expected values obtained under the geocentric axial dipole hypothesis) in time-averaged fields. (**a**–**d**) Anomalies derived from the palaeomagnetic field models shown in Fig. 1 covering time intervals given below model names. (**e**, **f**). Anomalies derived from heterogeneous simulations shown in Fig. 3 covering durations given. |Dec_MAX_ (*λ* = 0)| denotes the maximum absolute declination obtained at the equator since declination becomes arbitrarily large at high latitudes. |Δinc_MAX_| denotes the maximum Inclination Anomaly obtained globally, defined as measured inclination minus the inclination expected from the geocentric axial dipole hypothesis. Note that inclination shallowing produces a negative inclination anomaly in the Northern Hemisphere and a positive inclination anomaly in the Southern Hemisphere. Basemaps in **a**–**f** generated with M_Map^54^.

Our findings may prove important for studies of Earth’s ancient surface. Palaeomagnetism provides a major constraint on palaeogeographical reconstructions; persistent non-dipole field structures introduced by core-mantle heterogeneity may be sufficiently large to bias some of these. Tectonic reconstructions assume a geocentric axial dipole (GAD) time-averaged field morphology with any deviations typically assumed to affect only the palaeomagnetic inclination and to vary only in latitude^35^. Here we find that reflecting the influence of core–mantle heterogeneity, anomalies in both inclination and declination may exceed 10° and can vary in latitude and longitude (Extended Data Fig. 7). Further study of how the ancient geodynamo was impacted by mantle heterogeneity therefore offers the opportunity not only gain to insight into the structure and evolution of the lowermost mantle but also to inform on various longstanding debates^51–53^ surrounding Earth’s palaeogeography.

## Methods

### Palaeomagnetic datasets

Palaeomagnetic datasets used in this study each represent an age interval and are drawn from prior publications as detailed in Extended Data Table 1. Each dataset consists of a number (*N*_localities_) of localities (or studies): geographically restricted (typically < 100 × 100 km) regions comprising *N* site-mean palaeomagnetic measurements typically from single studies of rock units whose ages are within the bounds of the dataset. Site means within each locality are assumed to span sufficient time interval such that together, they provide a representative time-sampling of the palaeomagnetic field (~10^4^–10^6^ years). Localities were vetted in this respect (and in other respects relevant to them providing reliable estimates of the PSV) by the original publications. In this study, we take the further precaution of limiting the localities used to those with *N* ≥ 20. Each site mean itself comprises multiple individual specimen measurements, which are used to constrain the within-site dispersion (next section). Complete tables of all studies/localities used in this study are provided in Supplementary Dataset 1, and complete datasets are available on the MagIC database (Data Availability Statement). PSV10-24^35^ contains the latest in a series of updates^25,33^ of palaeomagnetic sites with ages < 10 Ma derived from volcanic units globally that have not been subject to post-emplacement deformation. The entire dataset contains 2,441 site-mean directions from 90 studies, but our selection criteria reduced this to 1,654 from 40 studies. These criteria were: (1) an averaged age ≤ 5 Ma; (2) at least three specimens per site with an associated Fisher precision parameter^55^ of 30 or higher; (3) at least 20 sites per locality. PSVM is the Miocene (5–23 Ma) dataset^27^ and consists of 1,454 palaeomagnetic sites from volcanic units at 44 localities with ages between 5 and 23 Ma. After applying selection criteria (2) and (3) above to PSVM, 1,218 sites from 27 localities were retained.

### Measures of palaeosecular variation and the time-averaged field

PSV analyses made use of virtual geomagnetic poles (VGPs) calculated using time-instantaneous magnetic field directions and location data^56^. The angular dispersion (*S*) of *N* VGPs from a time series at a given location are calculated using^23^:

2 $$S={\left[\frac{1}{N-1}\mathop{\sum }\limits_{i=1}^{N}{{\Delta }_{i}}^{2}\right]}^{\frac{1}{2}},$$

where ${\Delta }_{i}$ is the angular distance of the *i*th VGP from the mean VGP position, which is calculated using Fisher^55^ statistics. Corrections for within-site dispersion (unnecessary where VGPs were derived from a model) followed a well-described process^26,57^.

Values of *S* were calculated after a variable cut-off^36^ was applied. Outlier VGPs were defined as those with Δ_*i*_ > 1.8*S* + 5° and excluded. Subsequently, *S* and values of ${\Delta }_{i}$ are recalculated such that further outliers could then be removed. This was performed iteratively until no more outliers were identified. We consider it important to reject outliers because *S* is not a robust statistic and therefore is prone to being strongly influenced by the presence (or not) of anomalous directions that may be due to excursions, reversal transitions or human error. In our analyses, we applied a variable cut-off^36^ to both data and simulations but also tested two common alternative approaches: applying no cut-off and applying a fixed cut-off of VGP colatitude 45°, on two datasets (Supplementary Fig. 5). All three approaches produced *S*_|30|iqr_ values that are high relative to those obtained, using the variable cut-off, from the (non-multipolar) simulations run with homogeneous boundary conditions (Fig. 5). This suggests that the main findings of this study are robust to the choice of cut-off. We also note that our favoured variable cut-off approach produced a value of *S*_|30|iqr_ that is intermediate between the two alternatives and has overlapping uncertainty bounds.

All outputs of palaeomagnetic field models and dynamo simulations were initially expressed as a time series of Gauss coefficients (${g}_{l}^{m}$ and ${h}_{l}^{m}$) of degree *l* and order *m* describing the spherical harmonic expansion of the magnetic potential expressed at a distance above the source equivalent to the height of Earth’s surface above the core-mantle boundary (CMB)^58^. The potential for transient behaviour at the start of a simulation was considered through examination of the time series of the energies and the magnetic field components. Initial time steps were excluded from further analysis up until the point at which the behaviour could clearly no longer be construed as anomalous with respect to the bulk of the time series. Assuming a scaling factor of one magnetic diffusion time ≈ 200 kyr (ref. ^59^), simulations were run for the equivalent of tens to thousands of kyr (Supplementary Table 1). Each simulation was sampled uniformly in time at several thousand instants leading to sampling intervals ranging from several years to several kyrs. Some simulations did exhibit excursions and/or polarity reversals and the outlier VGPs produced were dealt with using the variable cut-off^36^ in an identical manner to the palaeomagnetic data.

PSV analysis of palaeomagnetic field models and dynamo simulations followed the approach of generating synthetic palaeomagnetic datasets described in detail elsewhere^37^. In summary:

1. The time series of Gauss coefficients defining the model or simulation was truncated to degree and order 10.
2. At a single location at the height of Earth’s surface, the instantaneous magnetic field direction was defined at 500 time steps drawn at random (with replacement) from the full duration of the model or simulation.
3. The corresponding VGPs were calculated, flipped if necessary to be in the Northern Hemisphere and then used to calculate *S* (using equation (2)) and the magnetic latitude (*λ*_m_; using equation (3)) at this location using
  3 $${\lambda }_{{\rm{m}}}=(\pi /2)-{\varDelta }_{s\to p}$$
  where *Δ*_*s→p*_ is the angular distance from the location to the mean VGP used in equation (2).
4. The above steps are repeated at locations across the globe with spacings of 10° in latitude and 20° in longitude.

Values of *S*_|30|median_ and S_|30|iqr_ are obtained in the same manner from palaeomagnetic datasets as from the outputs of models and geodynamo simulations. These use the *median* and *iqr* functions of MATLAB respectively and are defined as

4 $${S}_{|30{|}\mathrm{median}}={P\left({S}^{* }\right)}_{50}=\left\{\begin{array}{cc}{S}^{* }\left[\frac{n+1}{2}\right] & \mathrm{if}\,n\,\mathrm{is}\,\mathrm{odd}\\ \frac{{S}^{* }\left[\frac{n}{2}\right]+{S}^{* }\left[\frac{n}{2}+1\right]}{2} & \mathrm{if}\,n\,\mathrm{is}\,\mathrm{even}\end{array}\right.,$$

5 $${S}_{\left|30\right|\mathrm{iqr}}={P({S}^{* })}_{75}-{P\left({S}^{* }\right)}_{25},$$

where S* is the ranked collection of *S* values associated with *λ*_m_ ≤ ±30°; P(S*)_*x*_ is the *x*th percentile of this collection (obtained using linear interpolation between two individual ranked values of *S* if necessary) and *n* is the number of estimates of *S* in S*.

Where uncertainty estimates of *S*_|30|median_ and S_|30|iqr_ are provided (only for palaeomagnetic datasets because *N* is unconstrained in outputs from models), these reflect the 95% confidence range for the given parameter obtained from 10,000 bootstrap resamplings (performed with replacement) from the original dataset.

A value of 30° (N and S) for the upper bounds of the magnetic latitude window was chosen on the basis on the PSV10-24 results but we tested the robustness of the affected values derived from the PSVM dataset by varying it by ±5°. Our finding was that increasing this to 35° increased *S*_|30|median_ marginally but that S_|30|iqr_ was entirely unaffected by both changes (Supplementary Fig. 6).

We assessed the time-averaged fields output from palaeomagnetic field models and dynamo simulations following the process:

1. The time series of Gauss coefficients defining the model or simulation was truncated to degree and order 4, which represents the maximum degree to which the TAF is constrained. Note that a less severe truncation was required for the PSV because estimates of VGP dispersion may be derived directly from palaeomagnetic data that are not subject to regularization.
2. For time steps where the axial dipole term was positive, every Gauss coefficient at that time was multiplied by −1.
3. The arithmetic means of the Gauss coefficients from every time step were calculated and used to determine a set of time-averaged Gauss coefficients (not necessary for models LN3’ and MTAM1, which are already defined as a set of time-averaged Gauss coefficients)

Values of AD/NAD_TAF_ are then calculated using the Lowes power spectrum for the magnetic field energy (*W*)^60^ associated with these mean Gauss coefficients according to:

6 $${{\mathrm{AD}/\mathrm{NAD}}_{\mathrm{TAF}}=W}_{1}^{0}/(W-{W}_{1}^{0})$$

where

7 $$W=\mathop{\sum }\limits_{l=1}^{4}\mathop{\sum }\limits_{m=0}^{l}{W}_{l}^{m}$$

and

8 $${W}_{l}^{m}=(l+1)\left[{{(g}_{l}^{m})}^{2}+{{(h}_{l}^{m})}^{2}\right]$$

Likewise:

9 $${\mathrm{ZND}/\mathrm{ND}}_{\mathrm{TAF}}=\mathop{\sum }\limits_{l=2}^{4}{W}_{l}^{0}/\mathop{\sum }\limits_{l=2}^{l=4}\mathop{\sum }\limits_{m=0}^{l}{W}_{l}^{m}$$

### Numerical geodynamo simulations

Simulations were set up and solutions obtained following the same method are as described in our previous work^61–65^. In brief, an incompressible Boussinesq fluid is rotated with angular frequency Ω in a spherical shell with a ratio of inner to outer core radii of 0.35. In all simulations, no-slip mechanical boundary conditions, an electrically insulating outer boundary (mantle) and a fixed-flux outer boundary condition for the buoyancy source were used. Fluid motion is driven, at least partially, by thermal convection in all cases with bottom heating representing the release of latent heat at the inner core boundary. In double-diffusive simulations, chemical convection acts alongside the thermal component. The compositional component has zero flux at the outer boundary (representing no mass exchange with the mantle), a fixed radial flux of light element at the inner boundary and a uniform internal mass sink that achieves mass conservation of the light element^66,67^. In all cases, the inner core boundary was electrically insulating but, as a check, two simulations were re-run with conducting inner cores to verify that similar results were obtained (Supplementary Fig. 7).

Where simulations employing lateral variations in the outer boundary heat flux were run, it was assumed that LLVPs are anomalously hot regardless of the role of chemistry in producing their anomalous seismic properties. In all these cases, a tomographic model^1^ provided the basis for the pattern, which is dominated by the LLVPs giving rise to two antipodal and equatorial negative flux anomalies beneath Africa and the Pacific. The magnitude of the heterogeneity is varied using the q* parameter defined in equation (1). The power spectrum of the boundary condition is shown in Supplementary Fig. 8 and is dominated by degree 2 as is the lowermost mantle in most seismic tomographic models^3^.

All thermal simulations are characterized by 4 dimensionless parameters: the Ekman number (*E*), Prandtl number (Pr), the magnetic Prandtl number (Pm) and the thermal Rayleigh number (Ra_T_), given by

10 $$E=\frac{\nu }{\Omega {h}^{2}}$$

11 $$\Pr =\frac{\nu }{\kappa }$$

12 $$\mathrm{Pm}=\frac{\nu }{\eta }$$

Here *ν*, *η* and *κ* are the momentum, magnetic and thermal/compositional diffusivities, respectively, *h* is the shell thickness and$delete$

13 $${\mathrm{Ra}}_{{\rm{T}}}=\frac{{\alpha }_{{\rm{T}}}g{T}^{{\prime} }{h}^{2}}{2\Omega \kappa },$$

*g* is gravity at the outer boundary. Double-diffusive simulations require two additional parameters: a compositional Prandtl number (${\Pr }_{{\rm{C}}}$) and compositional Rayleigh number (${\mathrm{Ra}}_{{\rm{C}}}$) given by

14 $${\Pr }_{{\rm{C}}}=\frac{\nu }{D},$$

15 $${\mathrm{Ra}}_{{\rm{C}}}=\frac{{\alpha }_{{\rm{C}}}g{C}^{{\prime} }{h}^{2}}{2\Omega {\rm{D}}},$$

Here *D* is the compositional diffusion coefficient, ${\alpha }_{{\rm{T}}}$ and ${\alpha }_{{\rm{c}}}$ are the thermal and compositional expansion coefficients, and ${T}^{{\prime} }$ and ${C}^{{\prime} }$ are, respectively, the outer boundary temperature and inner boundary compositional gradients. All double diffusive simulations use Pr_C_ = 10. Note that all double diffusive simulations are in the ‘top-heavy’ regime where both thermal and compositional fields are destabilizing (for example, refs. ^66,68^) and so fingering and oscillatory instabilities do not arise in our simulations.

The new collection of geodynamo simulations presented here are subdivided into six groups within which only Ra_T_ (and Ra_C_ in the double diffusive case) were varied. In summary, the groups are as below with full details of all models used given in Supplementary Table 1:

1. THOM1: this sequence comprised thermally driven simulations with *E* = 3 × 10^−4^; Pr = 1; Pm = 3; q* = 0. A total of seven models were run with Ra_T_ varying in the range 120 to 1,750; a model with Ra_T_ = 100 was also run but proved submarginal for dynamo action.
2. THET1: this sequence of 11 simulations used identical parameters to THOM1 above with the exception that q* was raised to 2.3, and the range of Ra_T_ ran from 120 to 2,415.
3. TCHET1: this sequence of seven simulations was the double diffusive thermochemical equivalent to THET1 keeping the same values for all parameters except Ra_T_ and Ra_C_. The mass diffusivity was an order of magnitude lower than both momentum and thermal diffusivities giving Pr_C_ equal to 10. Ra_T_ and Ra_C_ were kept in a constant proportion of 1:50 such that the least strongly driven simulation had Ra_T_ = 150 and Ra_C_ = 7,500 and the most strongly driven Ra_T_ = 1,200 and Ra_C_ = 60,000.
4. THOM2: a pair of thermally driven simulations that were reported elsewhere^11^; the input parameters were *E* = 2 × 10^−5^; Pr = 0.2; Pm = 1, q* = 0, with Ra_T_ = 2,000 or Ra_T_ = 6,000.
5. THET2: as per THOM2 above but with q* = 2.3.
6. THET3: as per THOM2 and THET2 above but with q* = 5.0.

Parameters across the groups were chosen to maximize the opportunity to test the robustness of the results as manifested in the simulated palaeomagnetic behaviour they generated under different values of q*. Our simulations in groups THOM2, THET2 and THET3 used extreme parameters that were chosen to produce, as considered appropriate for the geodynamo^47,49^, higher values of magnetic energy (*E*_M_) than kinetic energy (*E*_K_) and magnetic Reynolds numbers (Rm) as close to 1,000 as possible (Supplementary Table 1). In THET3, q* was increased from 2.3 to 5.0 allowing a further test on the robustness on the degree of heterogeneity. Our choice of less extreme parameters for other simulations reduced computational expense allowing them to provide longer-duration runs in larger groups that sampled wide ranges of Ra more intensively while retaining the same q* values as THOM2 and THET2. Furthermore, using similar parameters, we were able to perform simulations (TCHET1) that used more Earth-like double-diffusion (also with q* = 2.3). This provided a further test of robustness by enabling us to assess thermochemical dynamics with the same heterogeneity enforced only on the thermal field. Whereas the very different input parameters for the two sets of simulation groups does not allow for easy comparison or the identification of trends, it does allow the universal features of their behaviour to be considered more robust than if a smaller area of parameter space was sampled.

### Magnetic screening analysis

A magnetic screening was applied to the output of simulations THOM2-60 and THET3-60 (Extended Data Fig. 6). The Gauss coefficients of each time step of these simulations were downward continued to the CMB and transformed into real space coordinates. LLVP shapes were based on the 1% slow contour^1^ and the magnetic field values within these regions were multiplied by a factor « 1. The magnetic field was then fit with a set of Gauss coefficients (again with degree and order 10) and upward continued back to Earth’s surface for each time step. The PSV assessed before and after this process (Extended Data Fig. 6) allowed the effects of screening to be isolated. The similarity of the VGP dispersion curves produced by THET3-60 supported that this simulation was already subject to such a screening effect. Similarly, a strong longitudinally varying pattern was imposed on the PSV output of THOM2-60 supporting the role of the screening process in generating this characteristic feature of heterogeneous simulations. Nevertheless, the spatially averaged PSV behaviour of THOM2-60 remained very different to that exhibited by its heterogeneously forced counterpart (THET3-60). This demonstrates that the screening effect in isolation, while considerable, is not sufficient to explain all the differences observed.

## Online content

Any methods, additional references, Nature Portfolio reporting summaries, source data, extended data, supplementary information, acknowledgements, peer review information; details of author contributions and competing interests; and statements of data and code availability are available at 10.1038/s41561-025-01910-1.

## Supplementary information

Supplementary Information Supplementary Figs. 1–8 and Table 1.

Supplementary Data 1 Supplementary Dataset 1.

## Source data

Source Data Fig. 1 Plotted PSV outputs/data and time-averaged Gauss coefficients.

Source Data Fig. 2 Plotted PSV outputs and time-averaged Gauss coefficients.

Source Data Fig. 3 Plotted PSV outputs and time-averaged Gauss coefficients.

Source Data Extended Data Fig. 4 Plotted summary data before and after downsampling.

## Figures and Tables

**Extended Data Table 1:**
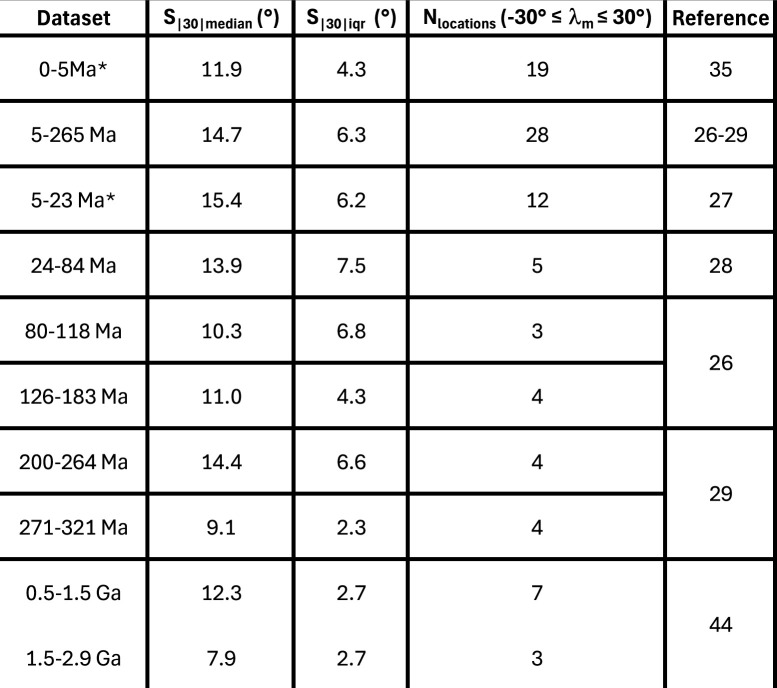
Palaeomagnetic datasets summarized in Fig. 5

Palaeomagnetic datasets summarized in Fig. 5. Dataset names correspond to the age range of the estimates of S from each of the references given. *S*_|30|median_ and *S*_|30|iqr_ are defined in the text. *N*_locations_ refers to the number of localities associated with absolute magnetic latitude of less than 30°. Each locality contributes an estimate of S from a minimum of 20 palaeomagnetic sampling sites. See Supplementary Dataset 1 for details of all localities. Individual localities are plotted for datasets marked with * in Fig. 1.

## Data Availability

All palaeomagnetic datasets are available on the MagIC database (www.earthref.org/MagIC/) (refs. ^69–73^). Results of all PSV and TAF analyses are available via figshare at 10.6084/m9.figshare.30849776 (ref. ^74^). Time-averaged field models are available on https://earthref.org/ERDA/2768/ and https://earthref.org/ERDA/2769/. Source data are provided with this paper.

## Code availability

Code for calculating the four parameters and reproducing associated plots and maps in Figs. 1–3, from any time series of Gauss coefficients, are available at via figshare at 10.6084/m9.figshare.30763625 (refs. ^75^).
