# Supplementary material for: Mantle heterogeneity influenced Earth’s ancient magnetic field
Source: Nat Geosci. 2026 Feb 3;19(3):345–52. doi: 10.1038/s41561-025-01910-1 (PMC12982131; doi:10.1038/s41561-025-01910-1)
Supplement: Supplementary file 1 — Supplementary Figs. 1–8 and Table 1. [file 41561_2025_1910_MOESM1_ESM.pdf]

nature geoscience

Supplementary information https://doi.org/10.1038/s41561-025-01910-1

Mantle heterogeneity influenced Earth's ancient magnetic field

In the format provided by the authors and unedited

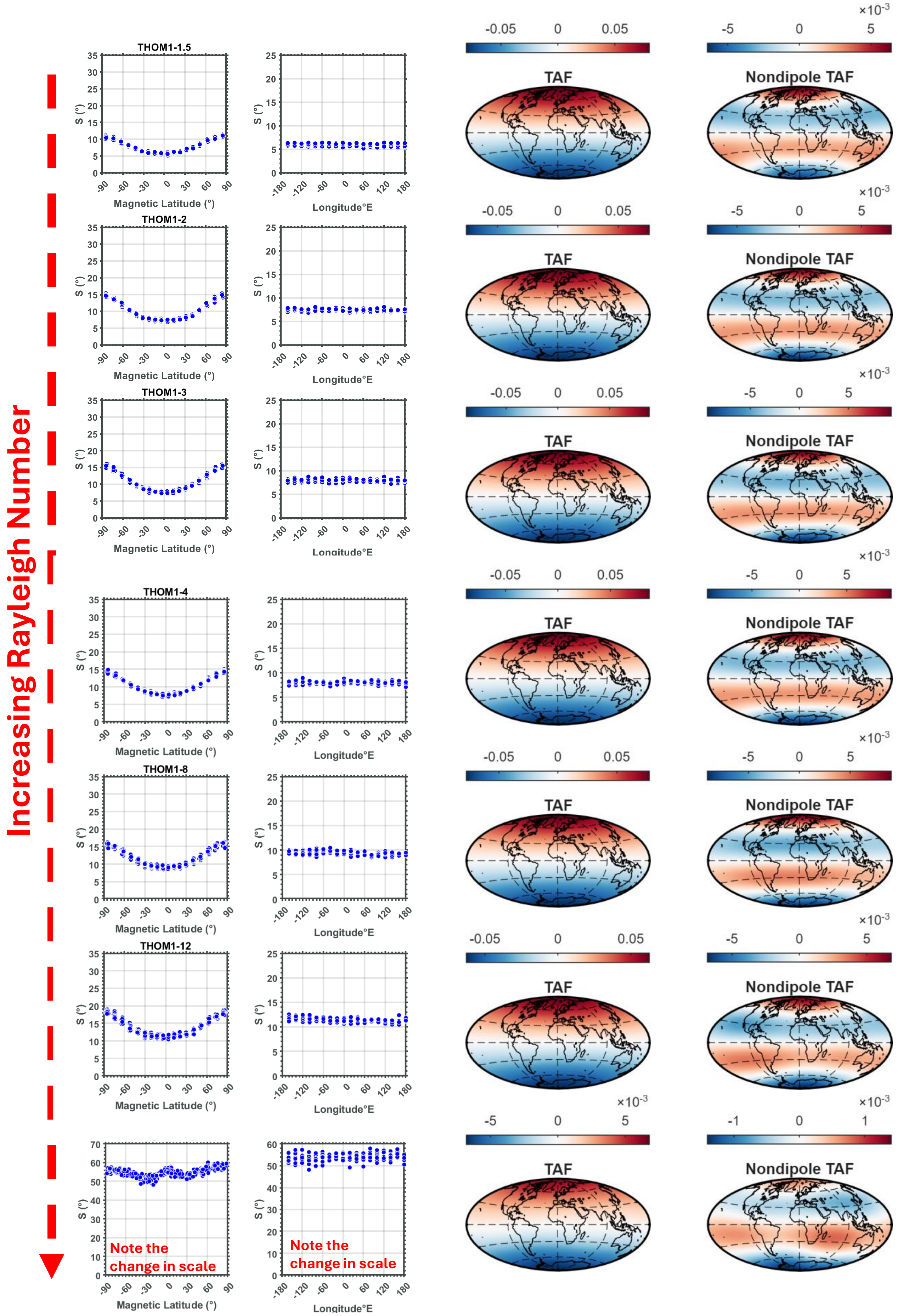

Supplementary Figure 1: Palaeosecular variation (PSV) and time-averaged field (TAF) output from THOM1 dynamo simulations (thermally driven with homogeneous outer boundary conditions). See Table S1 for input parameters of models and Figure 1 for descriptions of panels. All units are dimensionless unless specified. The M_Map tool[54] was used for mapping.

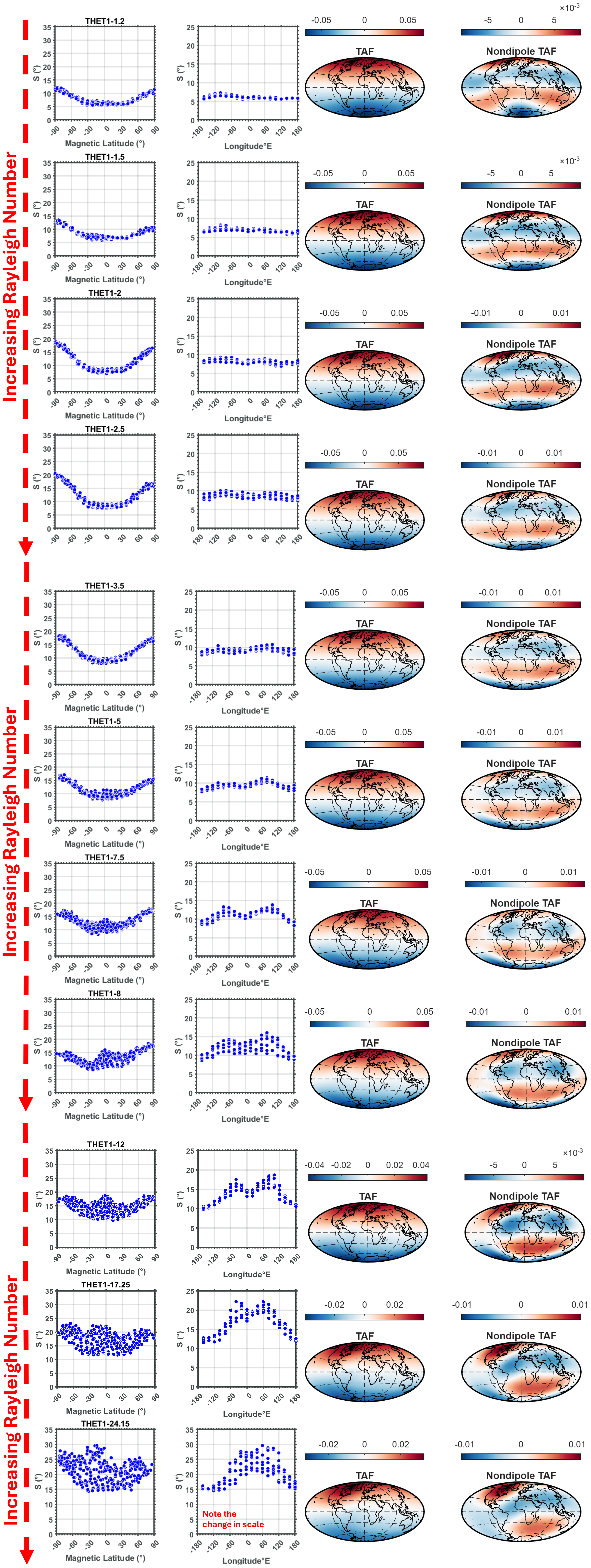

Supplementary Figure 2: Palaeosecular variation (PSV) and time-averaged field (TAF) output from THET1 dynamo simulations (thermally driven with heterogeneous outer boundary conditions). See Table S1 for input parameters of models and Figure 1 for descriptions of panels. All units are dimensionless unless specified. The M_Map tool[54] was used for mapping.

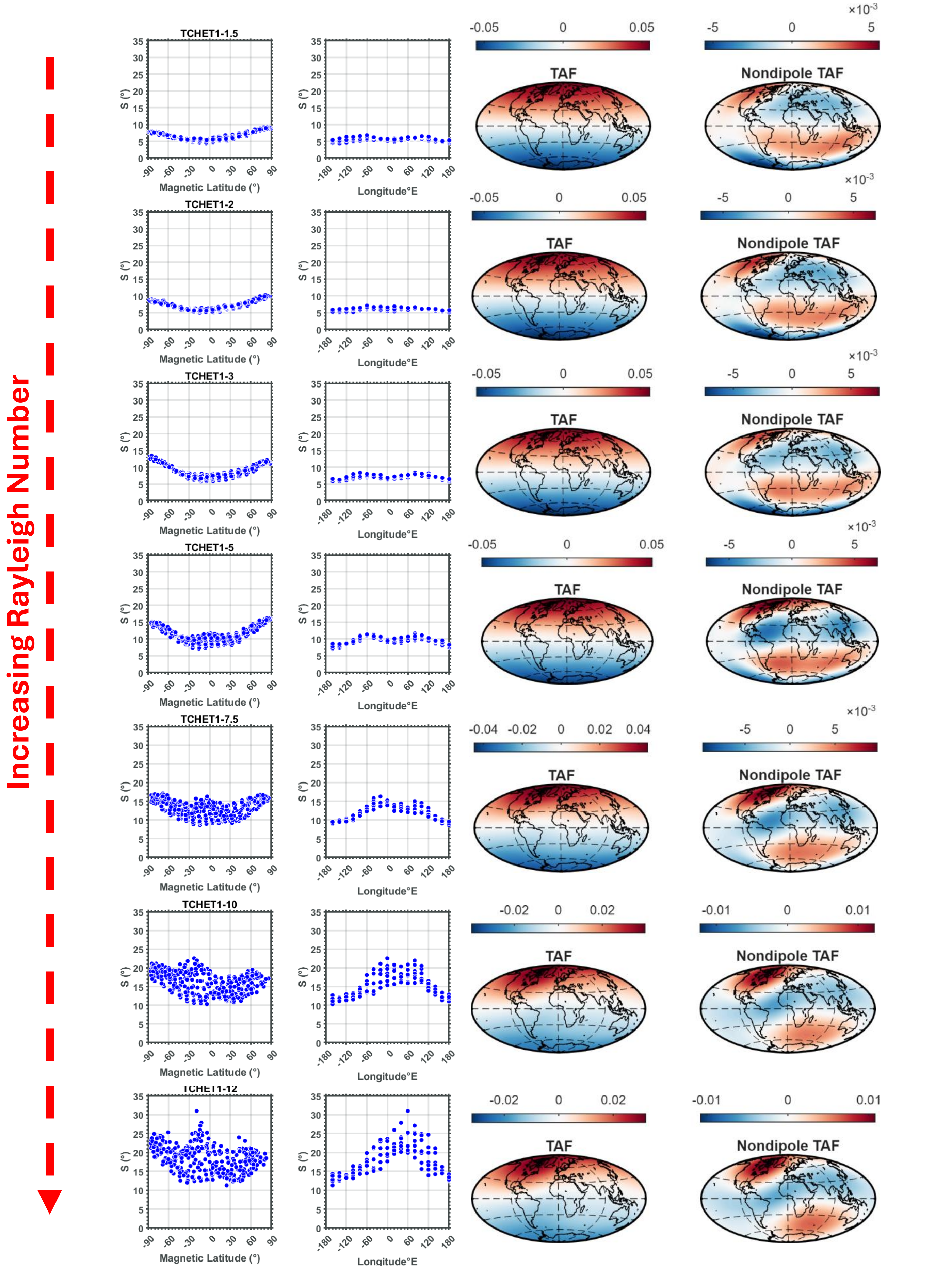

Supplementary Figure 3: Palaeosecular variation (PSV) and time-averaged field (TAF) output from TCHET1 dynamo simulations (thermochemically driven with heterogeneous outer boundary conditions). See Table S1 for input parameters of models and Figure 1 for descriptions of panels. All units are dimensionless unless specified. The M_Map tool[54] was used for mapping.

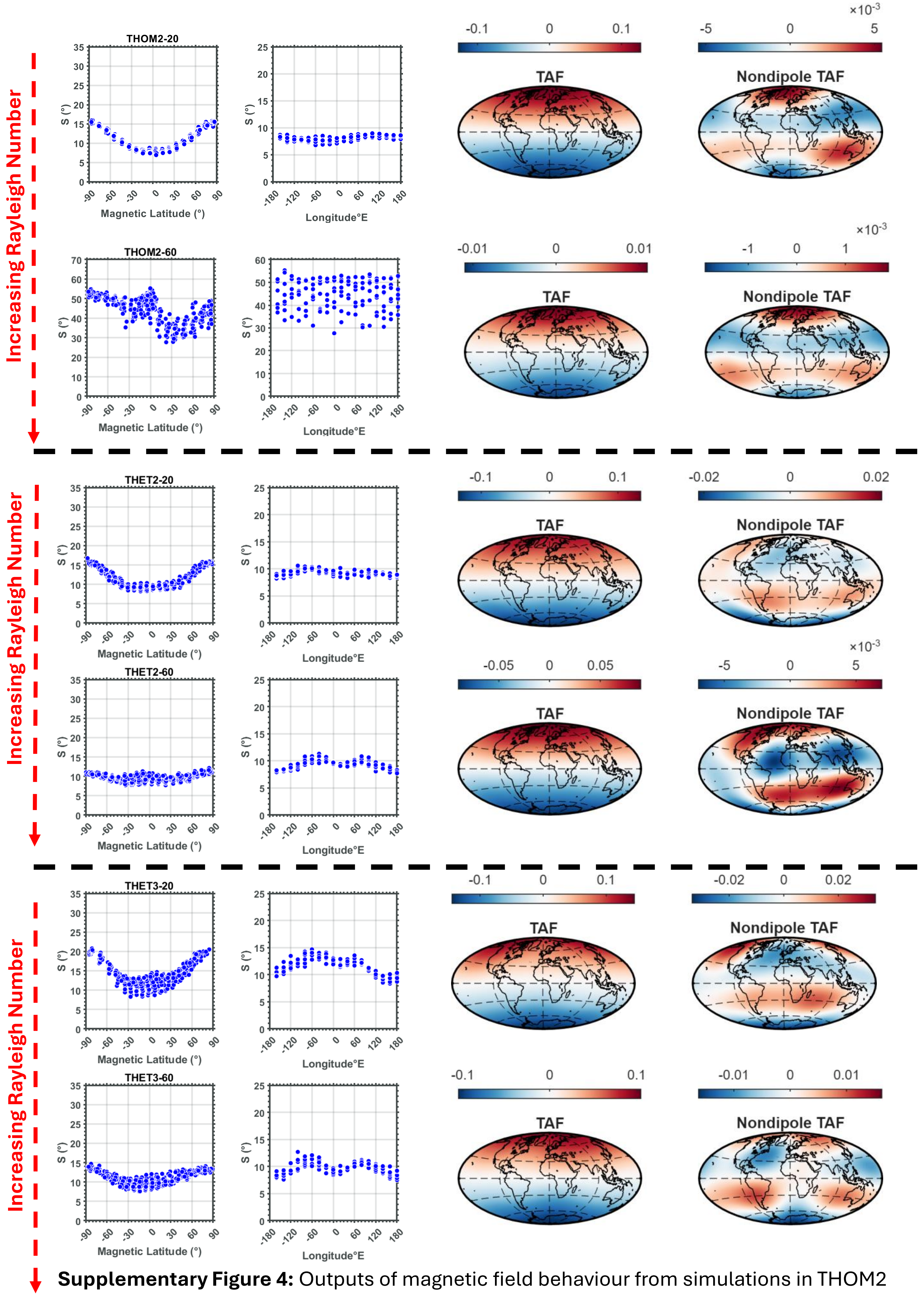

Supplementary Figure 4: Outputs of magnetic field behaviour from simulations in THOM2 (top), THET2 (middle), and THET3 (bottom). For description of panels, see figure 1. For model parameters, see Table S1. All units are dimensionless unless specified. The M_Map tool[54] was used for mapping.

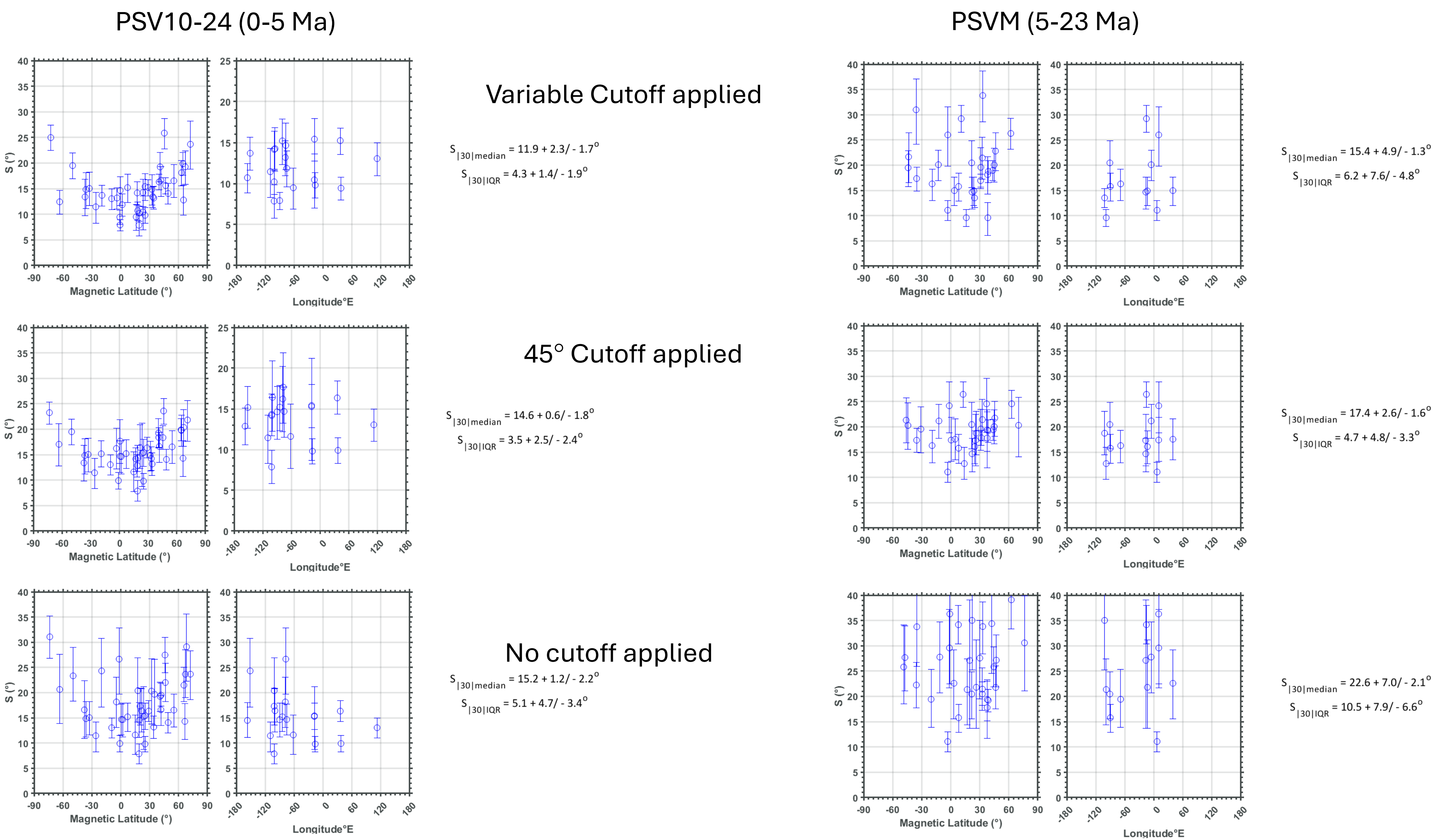

Supplementary Figure 5: Illustration of effects of applying different VGP cutoffs to the two main PSV datasets[27,35] used in this study. The variable cutoff was used throughout this study but parameters calculated using alternatives have overlapping uncertainty bounds with the exception of $S_{|30|median}$ for PSVM which is uniquely high. The data underlying all plots is identical and the same process (outlined in *Methods*) was followed for each except that a fixed cutoff of 45° or no cutoff was applied as indicated. S represents virtual geomagnetic pole dispersion as defined in *Methods* and error bars were calculated using 10,000 bootstraps with replacement.

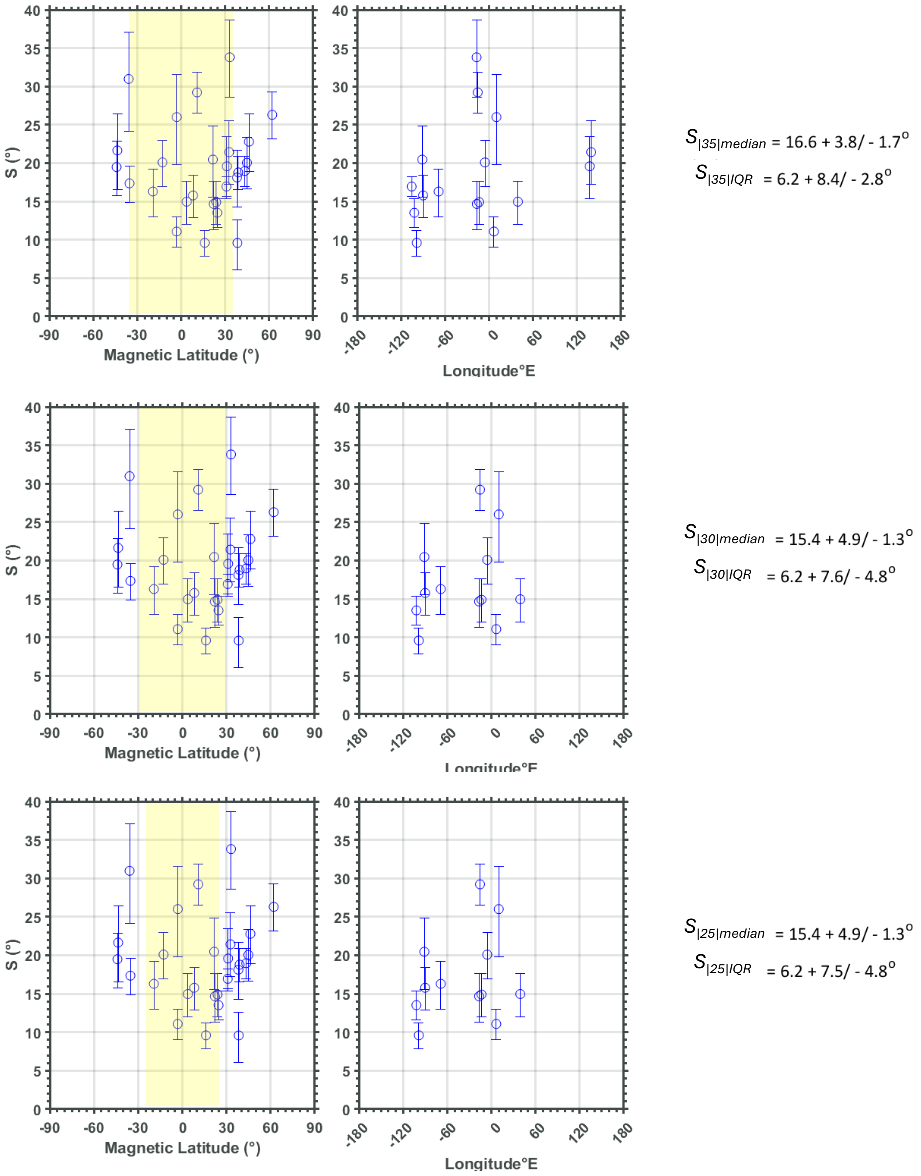

Supplementary Figure 6: Test of robustness for $S_{|30|median}$ *and* $S_{|30|IQR}$ for PSVM: the smallest, noisiest and, therefore, most at-risk dataset (Extended Data Table 1). Varying the latitudinal cutoff by ± *5°* is found to produce no significant impact on values. The data underlying all plots is identical and the same process (outlined in *Methods*) was followed for each except that the low latitude bounds were modified as indicated. S represents virtual geomagnetic pole dispersion as defined in *Methods* and error bars were calculated using 10,000 bootstraps with replacement.

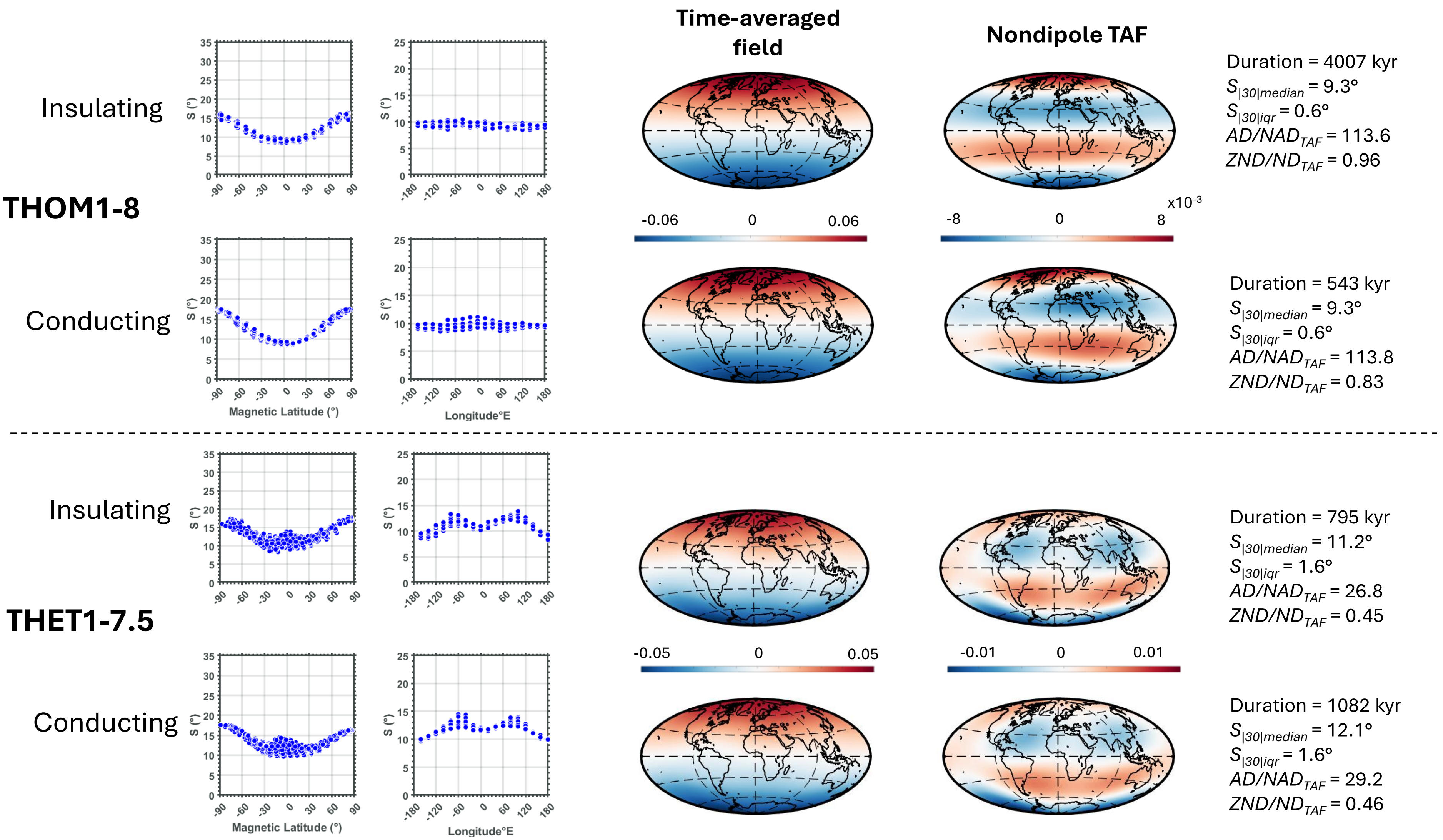

Supplementary Figure 7: Outputs of magnetic field behaviour from two simulations shown alongside simulations run with identical input parameters except the inner core was changed from electrically insulating to conductive. For description of panels, see figure 1. For model parameters, see Table S1. All units are dimensionless unless specified. The M_Map tool[54] was used for mapping.

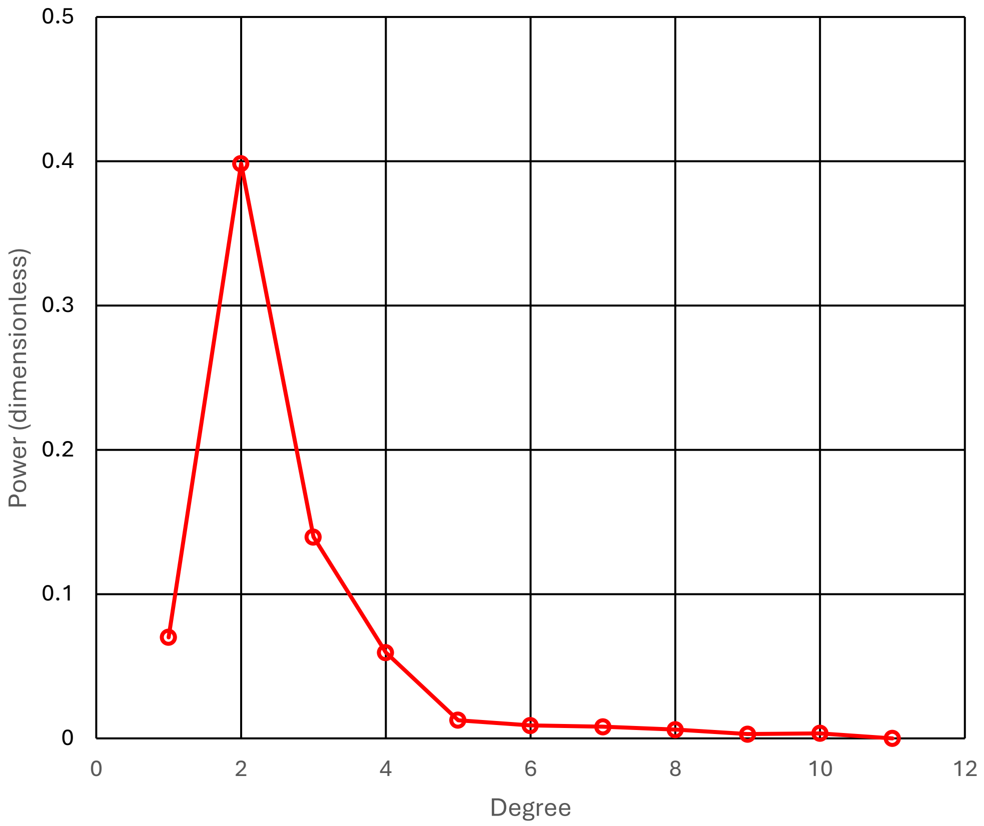

Supplementary Figure 8: Power spectrum of heat flow boundary condition used in the heterogeneous simulations.

Model	Reference	Group		Duration (kyr)	Ra_T	Ra_C	AD/NAD_{median}	f_{dip}	E_m/E_k	Rm	Ro	f_{ohm}	$S_{\|30\|median}$ (°)	$S_{\|30\|iqr}$ (°)	AD/NAD_{TAF}	ZND/ND_{TAF}
THOM1-1.5	This study	**THOM1** $q^* = 0$	Thermal $E = 3 \times 10^{-4}$ $Pr = 1$ $Pm = 3$	3426	150	0	42.8	0.84	2.54	107	0.011	0.37	5.9	0.4	289.1	0.99
THOM1-2	Me21			5688	200	0	26.4	0.75	3.42	121	0.012	0.43	7.4	0.3	178.7	1.00
THOM1-3	Me21			5110	300	0	22.4	0.70	2.98	148	0.015	0.43	7.9	0.4	147.8	0.99
THOM1-4	Me21			4345	400	0	22.5	0.69	2.27	176	0.018	0.41	7.9	0.5	130.1	1.00
THOM1-8	Me21			4007	800	0	16.7	0.63	1.16	262	0.026	0.35	9.3	0.6	113.6	0.96
THOM1-12	Me21			3156	1200	0	12.1	0.57	0.75	333	0.033	0.31	11.4	0.6	136.4	0.97
THOM1-17.5	This study			765	1750	0	0.2	0.15	0.17	724	0.072	0.19	53.8	2.5	36.0	0.92
THET1-1.2	This study	**THET1** $q^* = 2.3$		709	120	0	40.2	0.79	4.60	94	0.009	0.43	6.0	0.5	94.3	0.67
THET1-1.5	This study			2393	150	0	29.1	0.77	4.64	104	0.010	0.45	6.9	0.6	80.0	0.85
THET1-2	This study			2673	200	0	16.6	0.66	4.52	115	0.012	0.46	8.0	0.6	45.4	0.73
THET1-2.5	This study			708	250	0	14.1	0.64	3.84	148	0.015	0.45	8.4	0.9	45.2	0.83
THET1-3.5	This study			1230	350	0	13.8	0.63	2.61	156	0.016	0.40	9.1	0.7	40.1	0.67
THET1-5	This study			1718	500	0	13.7	0.61	1.76	205	0.021	0.36	9.3	1.1	40.6	0.62
THET1-7.5	This study			795	750	0	10.1	0.56	0.91	288	0.029	0.29	11.2	1.6	26.8	0.45
THET1-8	This study			182	800	0	9.4	0.53	0.91	274	0.027	0.34	11.9	2.9	28.0	0.44
THET1-12	This study			795	1200	0	7.9	0.50	0.60	340	0.034	0.27	13.9	3.6	24.2	0.32
THET1-17.25	This study			836	1725	0	5.7	0.46	0.46	389	0.039	0.26	16.8	5.7	19.2	0.29
THET1-24.15	This study			804	2415	0	3.5	0.38	0.34	496	0.050	0.24	20.7	7.0	11.6	0.29
TCHET1-1.5	This study	**TCHET1** $q^* = 2.3$	Thermochemical $E = 3 \times 10^{-4}$ $Pr = 1$ $Pm = 3$	771	150	7500	53.2	0.82	1.73	125	0.013	0.31	5.4	0.6	224.5	0.34
TCHET1-2	This study			870	200	10000	42.2	0.78	1.56	146	0.015	0.31	5.9	0.8	155.6	0.36
TCHET1-3	This study			983	300	15000	27.8	0.72	1.13	185	0.019	0.29	7.1	1.0	118.9	0.46
TCHET1-5	This study			497	500	25000	16.7	0.64	0.79	244	0.024	0.27	9.6	1.6	82.2	0.50
TCHET1-7.5	This study			432	750	37350	10.8	0.57	0.56	306	0.031	0.25	12.2	3.6	35.5	0.41
TCHET1-10	This study			480	1000	49800	5.3	0.48	0.45	357	0.036	0.24	15.5	5.5	12.7	0.38
TCHET1-12	This study			385	1200	60000	3.8	0.41	0.37	398	0.040	0.23	17.4	6.9	9.3	0.35
THOM2-20	MD23	**THOM2** $q^* = 0$	Thermal $E = 2 \times 10^{-5}$ $Pr = 0.2$ $Pm = 1$	121	2000	0	22.6	0.71	2.17	889	0.018	0.67	8.0	0.8	1008.3	0.76
THOM2-60	MD23			127	6000	0	0.5	0.19	0.41	1848	0.037	0.52	46.0	10.3	52.6	0.90
THET2-20	MD23	**THET2** $q^* = 2.3$		116	2000	0	18.5	0.64	2.69	851	0.017	0.68	9.2	0.7	109.6	0.48
THET2-60	MD23			131	6000	0	25.0	0.66	0.85	1608	0.032	0.59	9.4	1.2	173.8	0.47
THET3-20	MD23	**THET3** $q^* = 5.0$		53	2000	0	8.5	0.54	3.54	830	0.017	0.69	12.0	2.5	21.4	0.36
THET3-60	MD23			91	6000	0	17.5	0.63	1.41	1483	0.030	0.61	9.6	1.8	68.8	0.63

Supplementary Table 1: Summary of studied dynamo simulations. References Me21 and MD23 refer to refs 67 and 11 respectively. Group refers to definitions given in the Methods where q^* is the thermal heterogeneity on the outer boundary, E is the Ekman number, Pr is the Prandtl number, and Pm is the magnetic Prandtl number. Calculated durations assume a magnetic diffusion time of 200 kyr. Ra_T and Ra_C are the thermal and chemical Rayleigh numbers respectively. AD/NAD_{median} is the median of the time series of time-instaneous ratios of AD/NAD calculated at Earth's surface (ref 37; see Methods); f_{dip} is time-average dipolar fraction of the field at the core-mantle boundary (ref 69). E_m/E_k is the ratio of total magnetic and kinetic energies. Rm is the magnetic Reynolds number, Ro is the Rossby Number, and f_{ohm} is the fraction of Ohmic dissipation. Palaeosecular variation and time-averaged field parameters are given in the last four columns and are defined in the text.
